# Effect of acute exposure to moderate altitude on kinematic variables of the *ippon-seoi-nage* and its relationship with the countermovement jump in elite judokas

**DOI:** 10.1371/journal.pone.0206297

**Published:** 2018-10-24

**Authors:** Filipa Almeida, Juan Bonitch-Góngora, Paulino Padial, Blanca de la Fuente, Antonio J. Morales-Artacho, Belén Feriche

**Affiliations:** 1 Department of Physical Education and Sport, University of Granada, Granada Spain; 2 High Performance Center of Sierra Nevada, Spanish Sport Council, Granada, Spain; Virginia Commonwealth University Department of Internal Medicine, UNITED STATES

## Abstract

This study aimed to assess the effect of acute exposure to moderate altitude on kinematic variables of the *ippon-seoi-nage* and on the mechanical outputs of the countermovement jump (CMJ). Thirteen elite male judokas from the Spanish Judo Training Centre in Valencia (age: 21.54 ± 2.15 years) participated in the study. All of them performed an incremental CMJ test and an *ippon-seoi-nage* technique test before (N) and after the ascent to a moderate altitude of 2320 m above the sea level (H). A linear velocity transducer was attached to the bar to assess the mechanical outputs of each loaded CMJ at different percentages of their own body weight (25, 50, 75 and 100%). A wearable sensor was used to assess the kinematic variables (times, accelerations and angular velocities) transferred to a dummy during the technique test. The kinematic variables showed great individual reliability (CV = 8.46% in N; CV = 8.37% in H), which contrasted with low reliability observed when the whole group was considered. The smallest important CV ratio (>1.15) showed that H caused changes in the reliability of the kinematic variables, with some variables becoming more reliable and others losing the reliability they had in N. H also caused small increments in peak velocity across all loads tested in the CMJ (+3.67%; *P*<0.05). In contrast, no changes in the kinematic variables were verified. In addition, there was no association between leg extension capability and the acceleration (*r* = -0.16 ± 0.19 in N; *r* = -0.24 ± 0.19 in H) or angular velocity (*r* = -0.19 ± 0.24 in N; *r* = -0.30 ± 0.26 in H) of the *ippon-seoi-nage*, nor was acute exposure to H found to affect this association (*P*>0.05). Differences between individual and within-groups CV confirm the individual adaptations that each judoka makes during this technique. Additionally, the CV ratio shows a change in the space-time pattern of the technique in H. Therefore, it would be necessary to include an adaptation period to adapt the technique after the ascent in altitude. Further studies are needed to confirm the relationship and transference from the velocity gains in CMJ during altitude training.

## Introduction

There is increasing evidence that acute exposure to moderate altitude improves explosive actions in basic strength exercises (bench-press, back squat and squat jump exercises) [1–3] and in sports activities such as sprints, jumps and throws [4–6]. Some authors suggest that this improvement may be due to a reduction in aerodynamic resistance in approximate proportion to the square of the velocity when cycling, running or throwing objects [4,6,7]. Modified motor unit recruitment patterns due to an increased anaerobic metabolism [8,9] could also be partly responsible for the improvements [10]. In addition, an increase in spinal excitability has been related to acute simulated hypoxia [11] and a greater increase in the Hoffmann’s reflex amplitude of the soleus muscle has been described at acute moderate altitude when compared with sea level [12], which can be related to a direct effect of hypoxemia on the supraspinal structures [10]. All these findings seem to indicate a possible additive effect between air pressure and composition that could positively affect explosive actions performed in moderate altitude, while simulated hypoxia does not display changes of interest [2,13] and may reproduce different responses [14].

Judo is a combat sport whose goal is to throw the opponent on his back, by applying explosive throwing techniques. These techniques last approximately 1 s [15] and require high muscle power both in upper- and lower-body muscle groups [16]. Strength in judo has been studied through the assessment of basic strength exercises (bench-press, back squat and squat jump exercises) [16,17], but their transference to technical performance has not been studied. Judo technique has been traditionally analysed through systematic observation and some authors have analysed it by recording performance through complex and expensive camera systems at different sampling rates [18–20]. Additionally, most of the studies focused on the *tori*’s (person who throws) performance, without analysing the effect of that performance on the *uke* (person who is thrown), which is the main goal of a throwing technique in judo. Moreover, there is no studies on the effect of acute exposure to moderate altitude on the quality of the technical performance, which is crucial when training in altitude condition is used.

Therefore, the aim of this study was to analyse the effect of an acute exposure to a moderate altitude on the kinematic variables transferred to a dummy during the *ippon-seoi-nage*, on the mechanical outputs of the *tori*’s countermovement jump (CMJ), and on the relationship between both kinds of variables. We hypothesised that (1) the acute altitude exposure would improve the CMJ output and (2) both, the altitude effect in leg extension capability and on the the kinematic variables of the *ippon-seoi-nage* will improve the performance of this technique.

## Methods

### Participants

Thirteen male judokas from the Spanish Judo Training Centre in Valencia (age: 21.54 ± 2.15 years; body mass: 87.72 ± 18.95 kg; height: 181.55 ± 9.27 cm; fat percentage: 11.91 ± 3.25%) participated in this study. The study was carried out at the end of a special preparation mesocycle. The participants were advised to maintain their usual habits and dietary intake during the study. The habitual time schedule of the participants was as follows: wake up at 8 am; breakfast at 8:30 am; physical conditioning training session (resistance training or metabolic training) from 10 to 11:30 am; lunch at 2 pm; snack at 5 pm; judo session (technical and tactical skills training) from 7 to 9 pm; dinner at 10 pm; bedtime at 11:30 pm. All participants had been practicing judo for at least 10 years. Their technical level ranged from first to third Dan black belt and all of them have been medallists in junior or senior National Championships in Spain, Dominican Republic or Georgia, five of them in junior or senior European Cups, five in Continental Opens, one in Gran Prix, two in junior Continental Championships, and one in junior World Championships. Participants were selected on the following criteria: having a national or international medal in judo in the current season, no chronic diseases or recent injuries that could compromise their performance and experienced in resistance training, including the loaded CMJ exercise, for, at least, two years. Participants were instructed to avoid any strenuous exercise for a minimum of two days preceding the testing session. They were informed about the study protocol and signed a written informed consent form prior to investigation. The study protocol was approved by the Institutional Review Board of the University of Granada and was in accordance with the Declaration of Helsinki. The individual in this manuscript photo has given written informed consent (as outlined in PLOS consent form) to publish his photo.

### Experimental design

**Fig 1:**
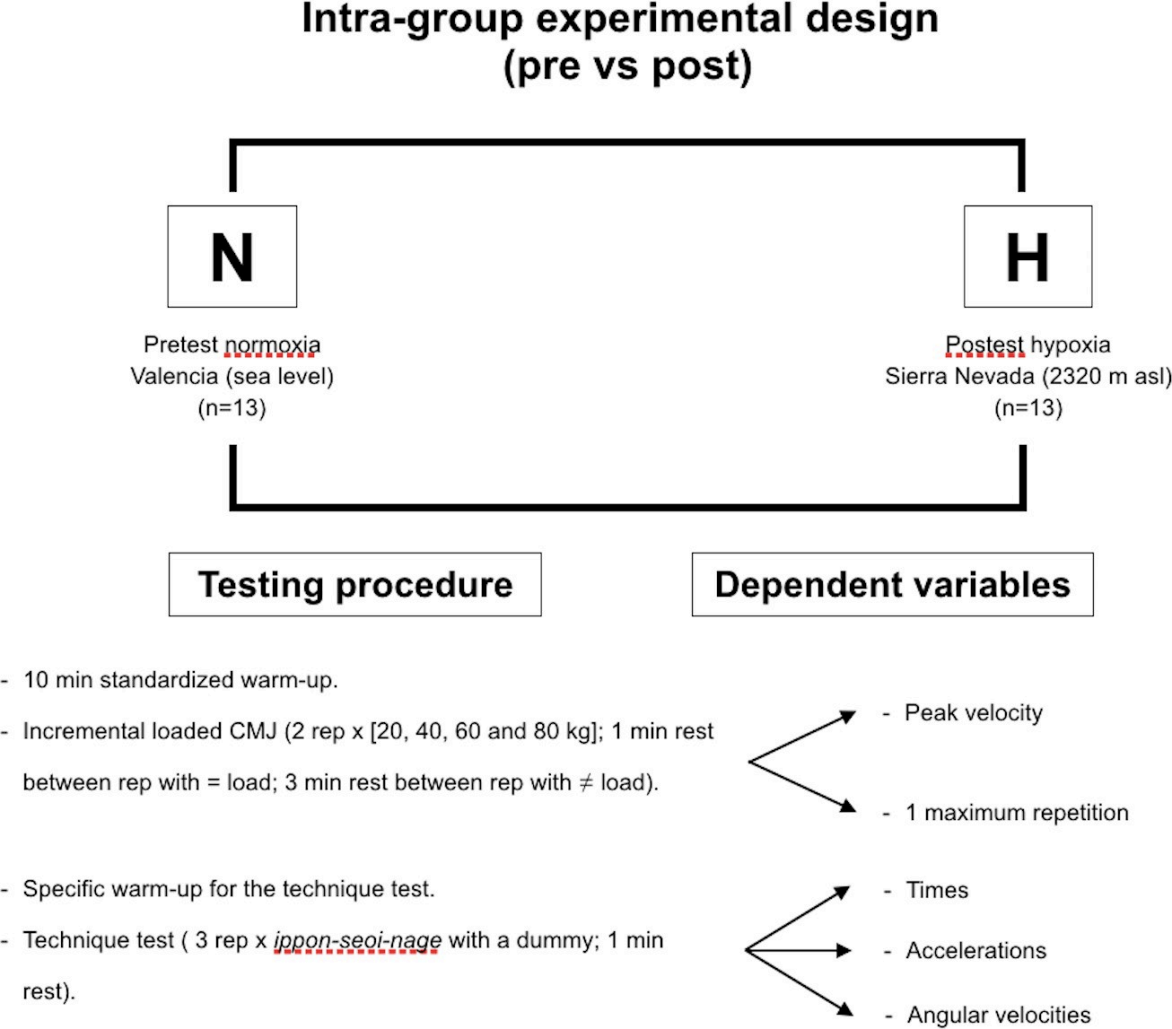
Experimental design.

The experimental design is shown in Fig 1. Peak velocity output (PV) and the 1 maximum repetition (RM) estimated from an incremental loaded CMJ test, and the kinematic variables transferred to the *uke* during the judo *ippon-seoi-nage* technique were assessed in both normoxia (N) and acute hypoxia (H) conditions. The H testing session took part immediately after the ascent to the High Performance Center of Sierra Nevada (Spain) at 2320 m above the sea level. The outcomes obtained under both environmental conditions were compared. Both testing sessions were conducted at the same time of the day for each participant and under the following environmental conditions: ~22° C and ~60% humidity or ~22° C and ~28% humidity, respectively for N and H conditions.

### Testing procedure

After a 10-minute standardized warm-up (jogging, dynamic stretching, joint mobility exercises, unloaded CMJs, and 5 CMJs loaded with 20 kg), participants undertook an incremental loaded CMJ test in a Smith machine. It consisted of 2 repetitions per each loading condition (20, 40, 60 and 80 kg), separated by 1 min of rest between repetitions with the same load and 3 min between different loading conditions. The repetition with the highest PV of each load was used for analysis. Participants performed the CMJ technique by standing with the knees and hips fully extended, feet approximately shoulder-width apart and the bar resting at the level of the acromion across the back. They were instructed to jump as high as possible after performing a countermovement to 90° of knee flexion. A manual goniometer was used to measure the 90° angle for each participant and an adjustable rod on a tripod was set with that individual height. In order to ensure the 90° knee flexion participants had to touch the rod with their glutei [21].

**Fig 2:**
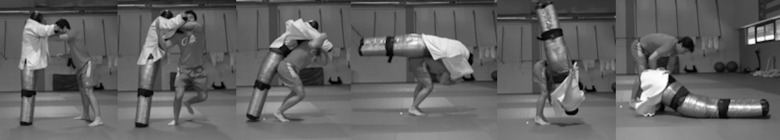
***Ippon-seoi-nage* sequence:** 1) starting position; 2) beginning of the repetition; 3) off-balance; 4) loading the dummy onto the back, reaching the dummy’s horizontal position and leg extension; 5) dummy’s flight over the judoka; 6) end of the repetition.

Afterwards participants performed a specific warm-up for the technique test (5 *ippon-seoi-nage* repetitions with a dummy [57 kg of weight]). The technique test started after 3 min of rest and included 3 repetitions of the *ippon-seoi-nage* with 1 min of rest between them. To perform this technique the judoka had to pull the dummy’s *judogi* (judo suit) to take it off-balance, turning to position himself underneath the dummy before finally throwing it over his shoulder (Fig 2). The judoka’s starting position was standing with feet shoulder-width apart, gripping the dummy’s *judogi*. Testing sessions were separated by a 72h-rest period.

### Data analysis and measurement equipment

Height (Seca 202, Seca Ltd., Hamburg, Germany) and body mass (Tanita BC 418 segmental, Tokyo, Japan) were assessed at the beginning of the testing session. The loaded CMJ was performed in a Smith machine (Multipower Fitness Line, Peroga, Murcia, Spain). A linear velocity transducer (T-Force System, Ergotech, Murcia, Spain) was attached to the bar of the Smith machine. The PV and mean propulsive velocity (MPV) of the bar during each jump was recorded at a 1000 Hz sampling rate. The relationship between load displaced and PV was established by fitting first-order-polynomials to the data. PV related to the load displacement equivalent to the 25, 50, 75 and 100% of the judoka’s body weight (PV25%BW, PV50%BW, PV75%BW and PV100%BW, respectively) was calculated through a regression equation. The 1RM was considered as the absolute load linked to a MPV of 0.33 m·s^-1^ obtained from the individual load-velocity relationships [22,23].

**Fig 3:**
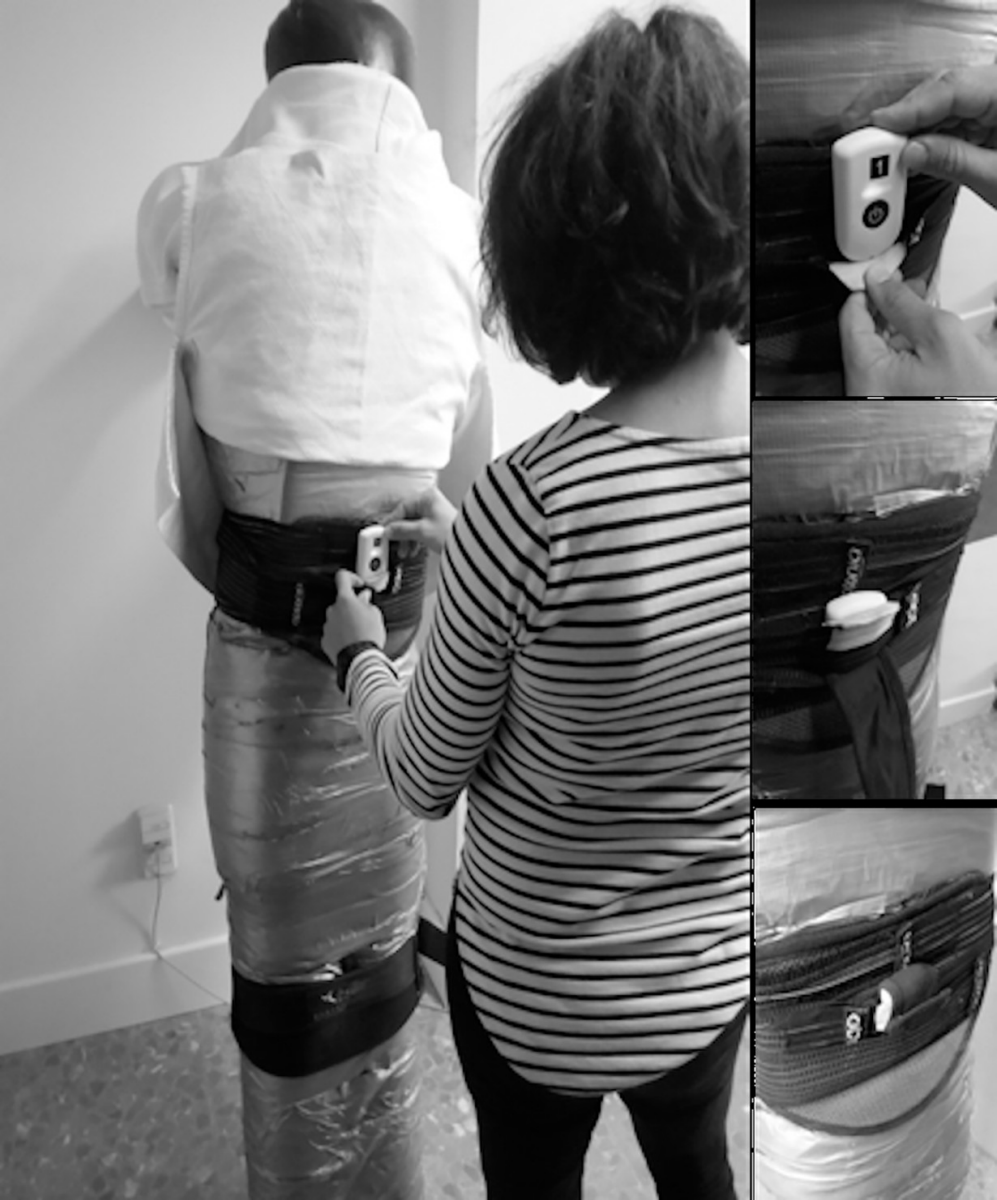
Placement of the wearable sensor (Wimu, Realtrack System, Almería, Spain) on the back of the dummy, fixed at waist height with a belt.

**Fig 4:**
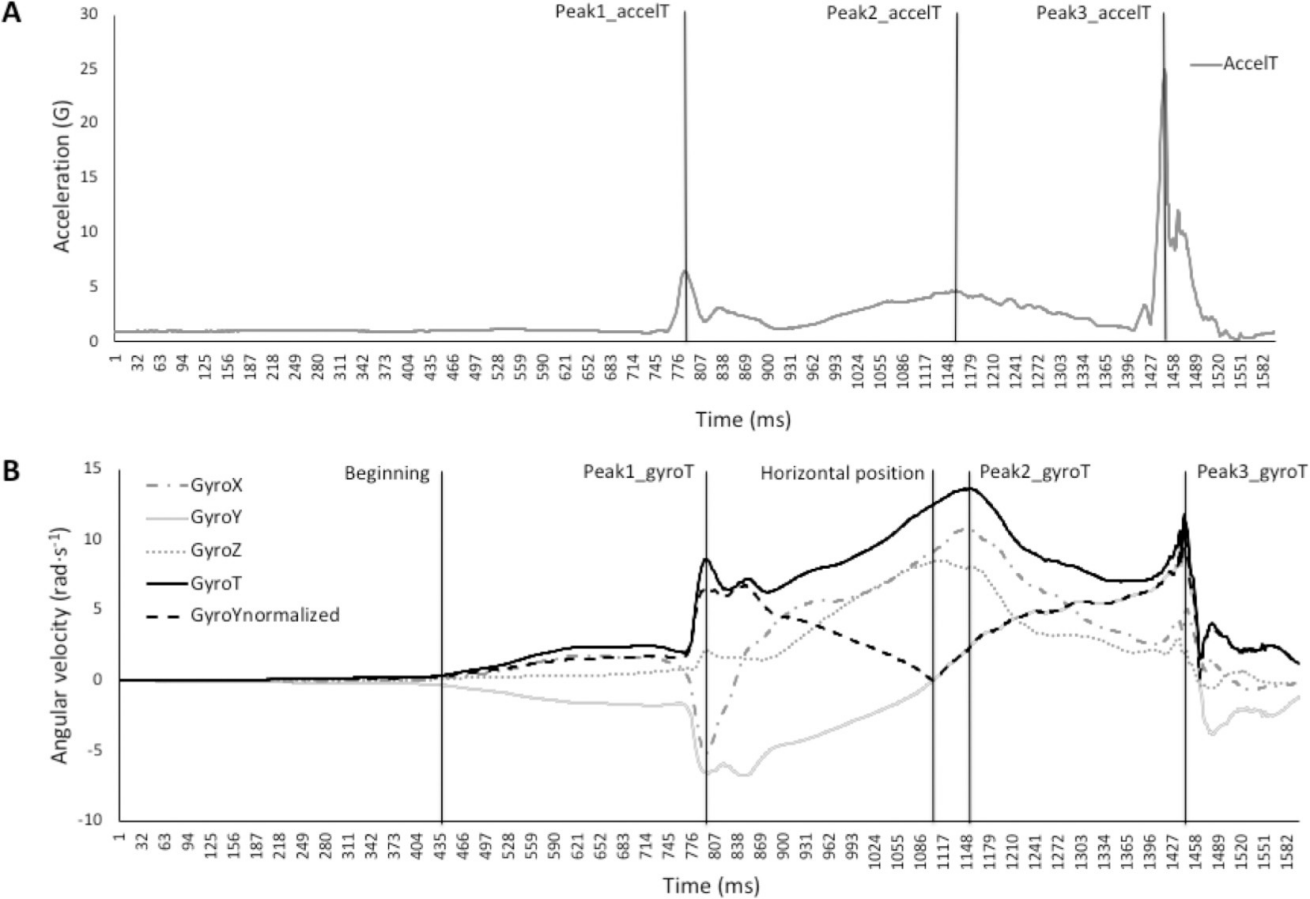
**Representation of the resultant acceleration (AccelT) (A), angular velocity in the three axes (GyroX, GyroY and GyroZ) and resultant angular velocity (GyroT) (B) of the *ippon-seoi-nage* performed by one judoka.** Three landmarks of the AccelT (Peak1_accelT, Peak2_accelT and Peak3_accelT) and of the GyroT (Peak1_gyroT, Peak2_gyroT and Peak3_gyroT) are displayed. The beginning of the repetition (considering the baseline of the GyroY) and the dummy’s horizontal position (represented by the inflection point on the GyroYnormalized) are also displayed.

Kinematic variables related to the dummy during the *ippon-seoi-nage* were recorded by using a wearable sensor (Wimu, Realtrack System, Almería, Spain) placed on the back of the dummy and fixed at waist height with a belt (Fig 3). This placement assured that the sensor was protected from direct impact from the *tori* or the floor and could be considered as the centre of mass. The device analysed acceleration (G) and angular velocity (rad·s^-1^) in the three axes (*x* or longitudinal axis, *y* or transversal axis and *z* or anterior-posterior axis) at a 1000 Hz sampling rate. The beginning of the repetition was defined as the moment when the dummy starts to become unbalanced (*i*.*e*., the angular velocity in the *y* axis deviates from the baseline with a permanent change of at least 50 ms). Three peaks of the resultant acceleration (AccelT) were determined, the first being related to the off-balance (Peak1_accelT), the second with the *tori*’s leg extension (Peak2_accelT) and the third with the dummy’s impact on the ground (Peak3_accelT) (Fig 4A). In addition, three peaks of the resultant angular velocity (GyroT) were assessed, the first related to pulling the dummy off balance (Peak1_gyroT), the second to the dummy’s flight over the *tori* (Peak2_gyroT) and the third to the end of the repetition (Peak3_gyroT) (Fig 4B).

The studied variables were organized as (1) time variables (T): time to reach the first, second and third peak of the resultant acceleration (Tpeak1_accelT, Tpeak2_accelT and Tpeak3_accelT, respectively), time to reach the first, second and third peak of the resultant angular velocity (Tpeak1_gyroT, Tpeak2_gyroT and Tpeak3_gyroT, respectively) and time to reach the dummy’s horizontal position (Thor); (2) acceleration variables: values of resultant acceleration in the first (Max1_accelT) and second peaks (Max2_accelT); and (3) angular variables: values of resultant angular velocity in the first (Max1_gyroT) and second peaks (Max2_gyroT).

A video camera Casio EX-F1 (Tokyo, Japan) was used to record the technical testing sessions at a 250 Hz sampling rate. Two experienced coaches rated the 3 *ippon-seoi-nage* repetitions based on the technical model approach of the Kodokan School [24]. The two best repetitions were selected for motion and reliability analysis. Afterwards the repetition with the lowest Thor was chosen as the best repetition for further analysis.

### Statistical analysis

Data are presented as mean ± standard deviation (SD). Normal distribution of the data was confirmed using a Shapiro-Wilk test. Between-repetition reliability of the *ippon-seoi-nage* kinematic variables was assessed by the within-subjects coefficient of variation (CV) and intraclass correlation coefficient (ICC) with their respective 95% confidence intervals. Additionally, the between-subjects CV and the individual CV were also calculated for each variable. An acceptable variability was defined as a CV < 15% and an ICC > 0.70 [25]. To interpret the magnitude of differences observed between the CVs from the 2 testing conditions (N vs H) a criterion for the smallest important ratio was established as higher than 1.15 [26]. Differences in the kinematic variables of the *ippon-seoi-nage*, PV output and estimated 1RM from the CMJ test between N and H were tested through a paired samples t-test. The magnitude of differences was expressed as standardized mean differences (Cohen’s effect size, ES). The criteria to interpret the magnitude of the ES was as follows: trivial (< 0.2), small (0.2–0.59), moderate (0.60–1.19), large (1.2–2.0) or very large (> 2.0) [27]. Correlation analysis between the kinematic variables of the *ippon-seoi-nage*, the PV output and estimated 1RM of the CMJ were assessed through a Pearson correlation coefficient (*r*) in N and H. The correspondent Fisher’s Z-transformed *r* coefficient was compared between N and H. The reliability analysis was performed by means of a custom spreadsheet [28], while SPSS software version 24.0 (IBM SPSS, Chicago, IL, USA) was used for all other analyses. Statistical significance was set at an alpha level of 0.05.

## Results

Tables 1 and 2 show the reliability of the *ippon-seoi-nage* kinematic variables in N and H conditions. The time, acceleration and angular variables displayed a good individual reliability in both conditions [CV = 8.46% (ranged from 4.58 to 12.83%) in N; CV = 8.37% (ranged from 4.2 to 14.4%) in H]. However, within-subject reliability remained within the standard minimum references (CV < 15% and ICC > 0.70) only in one of the time variables (Tpeak2_accelT), one of the acceleration variables (Max1_accelT) and one of the angular variables (Max1_gyroT) in N and in four of the time variables (Tpeak1_accelT, Tpeak3_accelT, Tpak1_gyroT and Thor) and two of the angular variables (Max 1_gyroT and Max2_gyroT) in H.

**Table 1:** Reliability of the kinematic variables of the *ippon-seoi-nage* in normoxia condition.

Variable	Repetition 1	Repetition 2	*P*	ES	Within-subjects CV (95% CI)	ICC (95% CI)	Between-subjects CV	Individual CV
Tpeak1_accelT (ms)	409.85 ± 79.22	423.00 ± 97.57	0.66	0.15	17.58 (12.61, 29.02)	0.35 (-0.22, 0.74)	21.23	10.87
Tpeak2_accelT (ms)	898.46 ± 132.55	933.08 ± 115.84	0.19	0.28	6.90 (4.94, 11.38)	0.78 (0.41, 0.93)	13.56	6.36
Tpeak3_accelT (ms)	1194.54 ± 99.36	1219.08 ± 115.80	0.47	0.23	6.92 (4.96, 11.42)	0.43 (-0.13, 0.79)	8.91	4.58
Max1_accelT (G)	6.10 ± 1.33	6.38 ± 1.86	0.44	0.17	14.25 (10.22, 23.53)	0.73 (0.33, 0.91)	25.58	12.14
Max2_accelT (G)	3.03 ± 0.83	2.75 ± 0.75	0.17	-0.36	17.52 (12.56, 28.92)	0.63 (0.15, 0.87)	27.40	12.83
Tpeak1_gyroT (ms)	431.85 ± 94.09	444.54 ± 101.76	0.67	0.13	16.94 (12.15, 27.97)	0.46 (-0.09, 0.80)	22.35	10.43
Tpeak2_gyroT (ms)	865.85 ± 130.43	908.31 ± 118.71	0.22	0.34	9.46 (6.78, 15.61)	0.59 (0.08, 0.85)	14.04	7.30
Tpeak3_gyroT (ms)	1173.92 ± 109.41	1197.15 ± 102.74	0.53	0.22	7.64 (5.48, 12.62)	0.30 (-0.28, 0.72)	8.95	5.20
Max1_gyroT (rad·s^-1^)	6.87 ± 1.02	7.10 ± 1.63	0.45	0.17	10.71 (7.68, 17.68)	0.73 (0.33, 0.91)	18.98	6.94
Max2_gyroT (rad·s^-1^)	10.35 ± 1.42	10.03 ± 1.36	0.41	-0.23	9.54 (6.84, 15.75)	0.55 (0.02, 0.84)	13.61	6.73
Thor (ms)	859.00 ± 122.18	896.15 ± 112.71	0.42	0.32	13.03 (9.35, 21.51)	0.06 (-0.49, 0.57)	13.38	9.73

*P*, *p-*value; ES, Cohen’s effect size; CV, coefficient of variation; ICC, intraclass correlation coefficient; 95% CI, 95% confidence interval; Tpeak1_accelT, Tpeak2_accelT and Tpeak3_accelT, time to reach the first, second and third peak of the resultant acceleration, respectively; Tpeak1_gyroT, Tpeak2_gyroT and Tpeak3_gyroT, time to reach the first, second and third peak of the resultant angular velocity, respectively; Max1_accelT and Max2_accelT, values of resultant acceleration that correspond to the peaks; Max1_gyroT and Max2_gyroT, values of resultant angular velocity that correspond to the peaks; Thor, time to reach the dummy’s horizontal position.

**Table 2:** Reliability of the kinematic variables of the *ippon-seoi-nage* in hypoxia condition.

Variable	Repetition 1	Repetition 2	*P*	ES	Within-subjects CV (95% CI)	ICC (95% CI)	Between-subjects CV	Individual CV
Tpeak1_accelT (ms)	405.31 ± 89.22	405.92 ± 83.85	0.97	0.01	10.74 (7.70, 17.73)	0.78 (0.42, 0.93)	21.33	9.06
Tpeak2_accelT (ms)	905.69 ± 151.13	891.00 ± 175.13	0.76	-0.09	13.54 (9.71, 22.35)	0.48 (-0.07, 0.81)	18.16	9.51
Tpeak3_accelT (ms)	1190.46 ± 153.29	1210.38 ± 162.38	0.54	0.13	6.71 (4.81, 11.07)	0.77 (0.41, 0.92)	13.15	5.14
Max1_accelT (G)	6.88 ± 1.62	6.97 ± 2.10	0.89	0.04	21.26 (15.25, 35.10)	0.41 (-0.15, 0.78)	26.80	13.04
Max2_accelT (G)	2.84 ± 1.09	2.56 ± 0.93	0.28	-0.28	23.29 (16.70, 38.45)	0.65 (0.18, 0.88)	37.40	14.40
Tpeak1_gyroT (ms)	419.15 ± 102.11	424.23 ± 103.46	0.78	0.05	10.72 (7.69, 17.69)	0.83 (0.54, 0.95)	24.37	9.32
Tpeak2_gyroT (ms)	903.77 ± 148.39	864.38 ± 132.69	0.28	-0.28	10.14 (7.27, 16.73)	0.63 (0.15, 0.87)	15.90	8.02
Tpeak3_gyroT (ms)	1149.77 ± 163.94	1180.08 ± 146.76	0.49	0.19	9.25 (6.64, 15.28)	0.56 (0.04, 0.84)	13.34	6.06
Max1_gyroT (rad·s^-1^)	6.99 ± 1.22	6.93 ± 1.17	0.68	-0.05	5.29 (3.79, 8.73)	0.92 (0.76, 0.98)	17.14	4.20
Max2_gyroT (rad·s^-1^)	10.20 ± 1.92	10.01 ± 1.55	0.63	-0.11	10.03 (7.19, 16.56)	0.70 (0.27, 0.90)	17.16	6.75
Thor (ms)	878.85 ± 175.44	879.23 ± 180.12	0.99	0.00	8.82 (6.32, 14.56)	0.84 (0.55, 0.95)	20.22	6.55

*P*, *p-*value; ES, Cohen’s effect size; CV, coefficient of variation; ICC, intraclass correlation coefficient; 95% CI, 95% confidence interval; Tpeak1_accelT, Tpeak2_accelT and Tpeak3_accelT, time to reach the first, second and third peak of the resultant acceleration, respectively; Tpeak1_gyroT, Tpeak2_gyroT and Tpeak3_gyroT, time to reach the first, second and third peak of the resultant angular velocity, respectively; Max1_accelT and Max2_accelT, values of resultant acceleration that correspond to the peaks; Max1_gyroT and Max2_gyroT, values of resultant angular velocity that correspond to the peaks; Thor, time to reach the dummy’s horizontal position.

**Fig 5:**
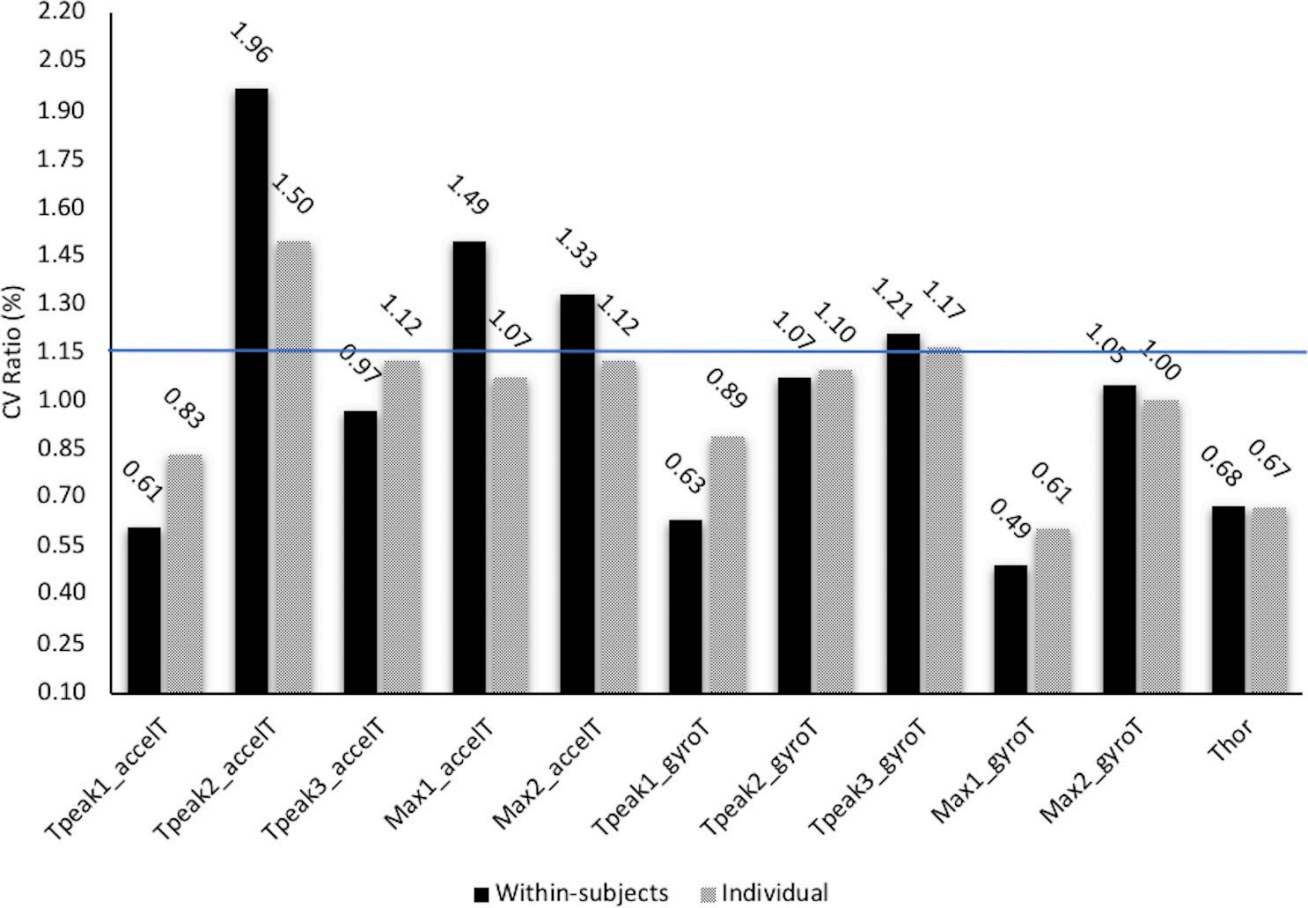
Reliability comparisons of the kinematic variables observed through the coefficient of variation (CV). The presented CV ratios are calculated as H/N. The smallest important ratio was considered to be 1.15.

The within-subjects CV ratio between H and N indicates that some variables were more reliable in N [Tpeak2_accelT (CV ratio = 1.96); Max1_accelT (CV ratio = 1.49); Max2_accelT (CV ratio = 1.33) and Tpeak3_gyroT (CV ratio = 1.21)]. Only two of the eleven CV ratios exceeded the individual CV ratio of 1.15 [Tpeak2_accelT (CV ratio = 1.50) and Tpeak3_gyroT (CV ratio = 1.17)] (Fig 5).

**Fig 6:**
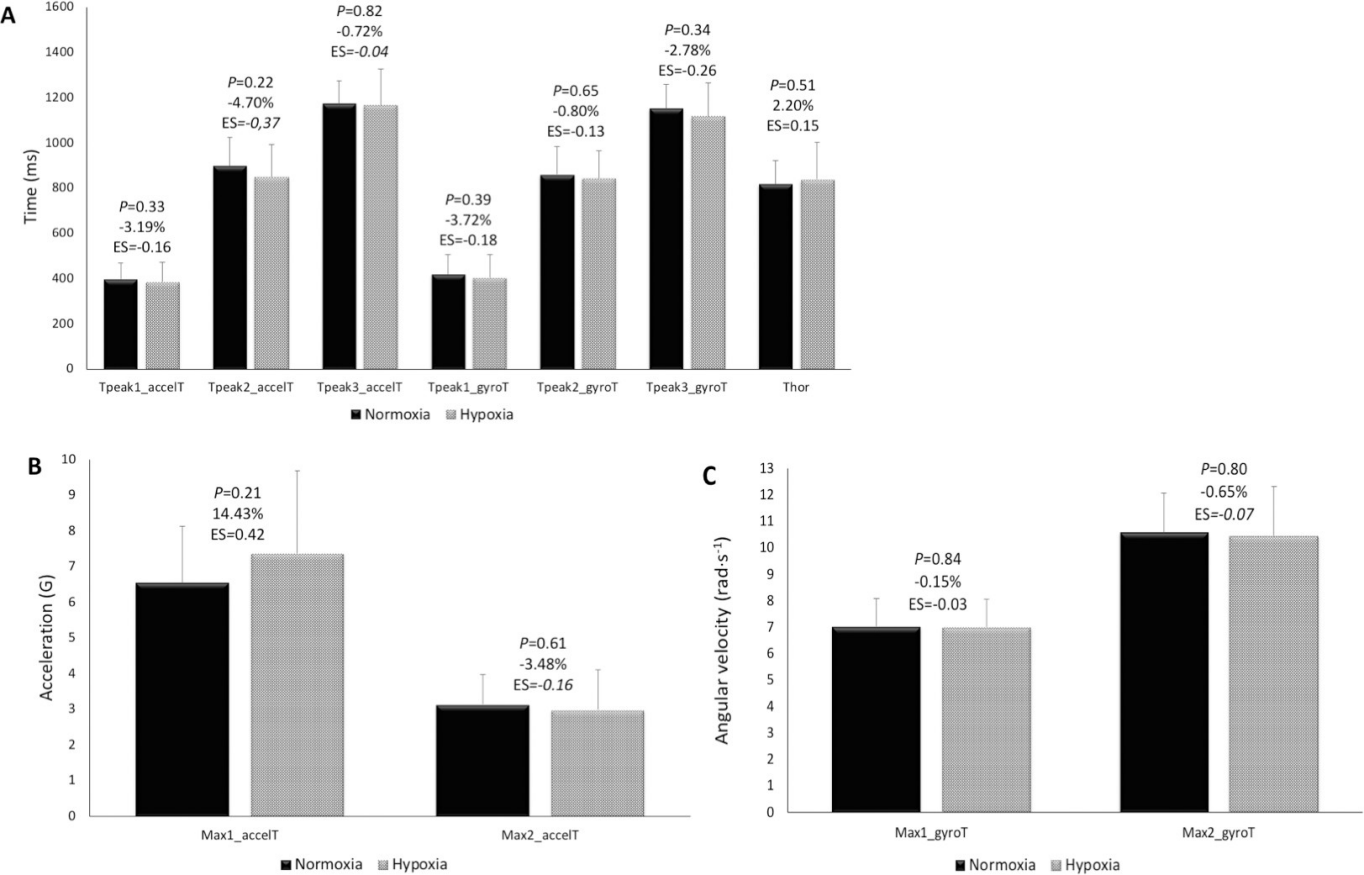
**Acute effect of hypoxia on the time (A), acceleration (B) and angular variables (C) of the *ippon-seoi-nage*.** No significant differences between normoxia and hypoxia (P > 0.05). The magnitude of differences was expressed as standardized mean differences (Cohen’s effect size, ES) and as percentage of change. Mean ± SD. Tpeak1_accelT, Tpeak2_accelT and Tpeak3_accelT, time to reach the first, second and third peak of the resultant acceleration, respectively; Tpeak1_gyroT, Tpeak2_gyroT and Tpeak3_gyroT, time to reach the first, second and third peak of the resultant angular velocity, respectively; Thor, time to reach the dummy’s horizontal position; Max1_accelT and Max2_accelT, values of resultant acceleration that correspond to the peaks; Max1_gyroT and Max2_gyroT, values of resultant angular velocity that correspond to the peaks.

**Fig 7:**
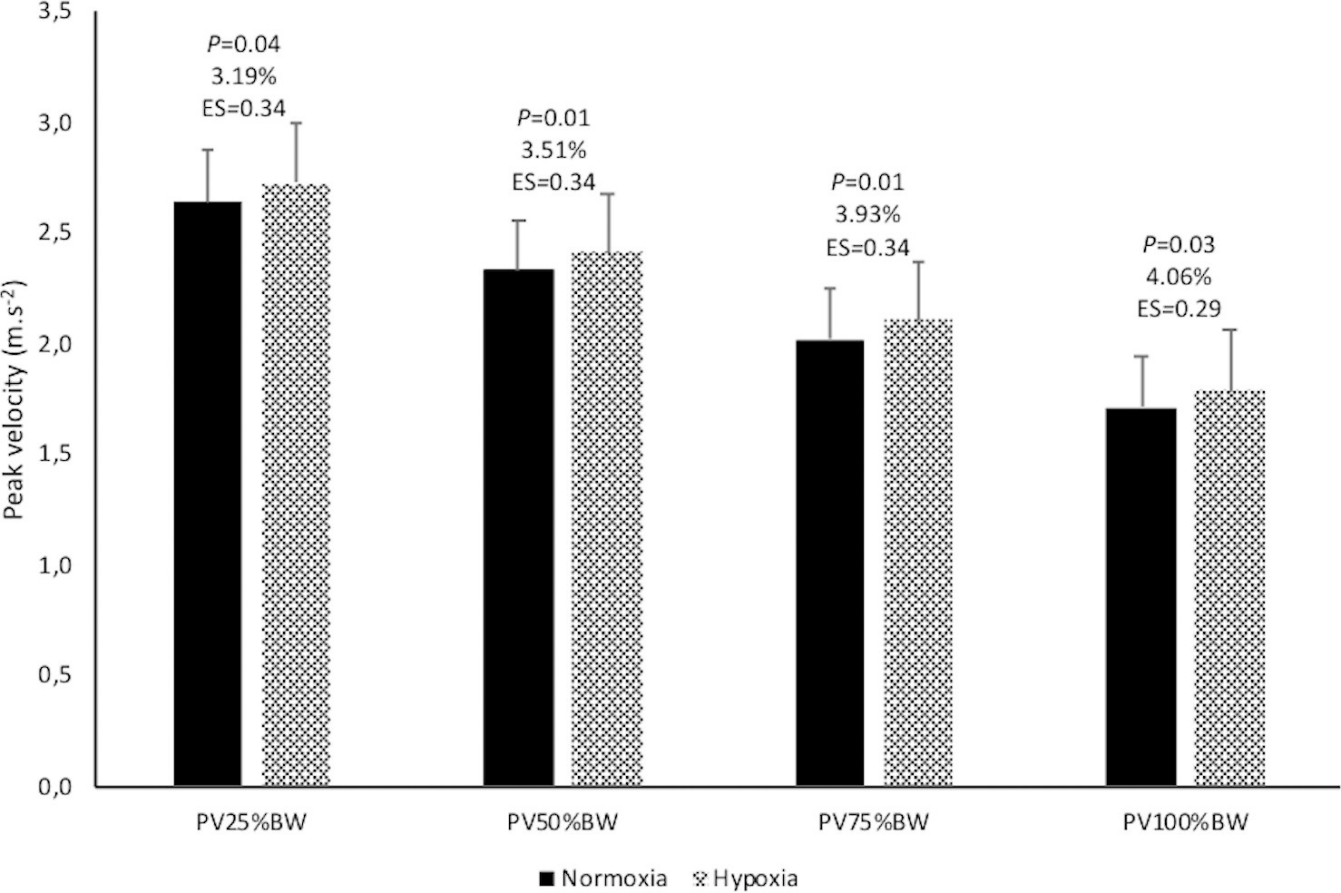
Acute effect of hypoxia on the peak velocities of the countermovement jump. There were significant differences between normoxia and hypoxia (P < 0.05). The magnitude of differences was expressed as standardized mean differences (Cohen’s effect size, ES) and as percentage of change. Mean ± SD. PV25%BW, PV50%BW, PV75%BW and PV100%BW, peak velocity with 25, 50, 75 and 100% of the judoka’s body weight, respectively.

Figs 6 and 7 show the comparative analysis results of the studied variables between N and H. The kinematic variables corresponding to the best technical repetition selected did not display any relevant change (Fig 6). Small increments in PV were reached during the loaded CMJ test in H conditions (+3.67%; *P* < 0.05) (Fig 7). No differences in 1RM relative to body weight (1.86 ± 0.31 vs 1.87 ± 0.37 kg·kg^-1^ for N and H, respectively; *P* = 0.86; ES = 0.02) were observed by the ascent to a moderate altitude.

No correlations were found between the Max2_accelT and the CMJ PV (*r* = -0.16 ± 0.19 in N; *r* = -0.24 ± 0.19 in H), nor between the Max2_accelT and the estimated 1RM of the CMJ (*r* = -0.37 in N; *r* = 0.09 in H). Similarly, the Max2_gyroT showed no correlations with the CMJ PV (*r* = -0.19 ± 0.24 in N; *r* = -0.30 ± 0.26 in H), nor with the estimated 1RM of the CMJ (*r* = -0.47 in N; *r* = 0.06 in H). The Fisher’s Z-transformed *r* coefficient comparison showed no differences between the correlations in N and H (*P* > 0.05).

## Discussion

The main aim of this study was to verify if the acute exposure to a moderate altitude affected the times, angular velocities and accelerations transferred to the *uke* during the performance of the *ippon-seoi-nage* in elite judokas and its relationship with their leg extension capacity. This is the first study that analyses the relationship between leg extension capacity and *ippon-seoi-nage* performance, as well as the altitude effect on the strength of this association and on the technical efficiency. The analysis of the kinematic variables of the *ippon-seoi-nage* shows a great individual reliability of the technique in both conditions, this contrasts with the low reliability observed when the whole group is considered. This result illustrates the difficulty in analysing a complex sporting technique such as the *ippon-seoi-nage*, reflecting particular adaptations applied by each judoka. Although acute H caused small increments in PV in the CMJ loaded with 25 to 100% of the judoka’s body weight, no changes in the kinematic variables were verified as a result of this. In addition, there was no association between the leg extension capability and the acceleration or angular velocity transferred to the uke, nor did acute exposure to H affect this association.

As has been shown in previous studies, acute exposure to moderate altitude improves explosive actions in basic strength exercises [1–3] and in sports activities [4–6]. This may be related to various factors like reduction in aerodynamic resistance [4,6,7], modified motor unit recruitment patterns due to an increased anaerobic metabolism [8,9], and a direct effect of hypoxemia on the supraspinal structures [10–12], which could justify the positive altitude effect on explosive exercise performance. The improvement in the PV of the CMJ observed in this study is in line with these studies, however the lack of improvement in the kinematic variables of the *ippon-seoi-nage* seems to contradict them, despite the explosive character of the movement and the great angular velocity and acceleration reached during the performance of *ippon-seoi-nage*. The duration of the *ippon-seoi-nage* observed (1.150 ± 0.108 s in N; 1.117 ± 0.149 s in H) is in accordance with the literature [15,29]. As stated by Bonitch-Domíguez et al. [16] judo techniques require high muscle power in lower-body muscle groups, and a judoka should be capable of applying this power, especially during the leg extension phase of the *ippon-seoi-nage* (represented in the structure of the movement by the Peak2_accelT, Fig 4A). The absence of association between the PV and 1RM of the CMJ with the Max2_accelT and Max2_gyroT could indicate that, at least in the sample studied, there is not a stability in the legs implication during the *ippon-seoi-nage*, which can be replaced or masked by the action of the arms, trunk or the turn itself. From a mechanical point of view, a rise in the PV during the CMJ should be linked to a rise in the acceleration and angular velocity in the peak 2 of the *ippon-seoi-nage*. Taken together, these findings indicate that further studies on the effect of a training program for the leg extension capacity and its transference to the technique are needed.

Results displayed a great individual reliability of the kinematic variables in both conditions while a low reliability was observed when the whole group was considered. This result shows that *ippon-seoi-nage*’s individual performance patterns coexist and are highly reproduced by each judoka, while at the same time, they significantly differ from the technique used by others. The individual adaptation of the *ippon-seoi-nage* may be caused by different factors, such as morphology [30], physical condition or competitive level [31]. The comparison between N and H has shown that after an acute ascent to a moderate altitude the reliability of the kinematic variables changed, and some variables became more reliable while others lost the reliability they had in N (Fig 5). The increase in the CV ratio of the time needed to reach the peak 2 of the acceleration (Tpeak2_accelT) suggests a certain change in the space-time pattern of the *ippon-seoi-nage* and is evidence of the need to adjust and stabilize the technique after the ascent to moderate altitude.

The individual reliability results of this study confirm the utility of the use of a high sampling rate wearable sensor in the kinematic analysis of complex techniques in judo. Most of the studies that analysed kinematic parameters of the *ippon-seoi-nage* focused on the variables of the *tori* [18,20,30] and the few that studied the *uke*’s motion [19] did not present data related with his resultant acceleration or angular velocity. To our knowledge, this is the first study to report direct acceleration and angular velocity measurements in the *ippon-seoi-nage* and may provide a useful reference for future comparisons.

The limitations of this study include the possible placebo effect. This problem is recurrent when the studies at a terrestrial altitude cannot be conducted single- or double-blind. However, while a psychological effect cannot be discarded in the present study, it is important to note that judokas were not aware or given any information about the effects of altitude exposure on performance. Additionally, the outcome of this study was limited by including only data concerning the *uke*’s motion. Consequently, it was difficult to fully determine the relationship between the *tori*’s movements and the *uke*’s resultant movements. A good relationship between leg extension capacity and technical perfomance was expected and the improvement of this capacity by altitude ascent has been previously described in several studies [1,3,10]. For this reason, it was of great interest to analyse if transference of this improvement happens in acute moderate altitude. However, we failed to demonstrate this association and future studies are needed to further analyse *tori*’s and *uke*’s motion relationship. Another limitation of the present study was that although every participant mastered the *ippon-seoi-nage*, it might be that this specific technique is not always their favourite to apply in competition despite it is one of the most used [20].

## Conclusions

High individual reliability of the *ippon-seoi-nage* kinematic variables in N and H contrasts with the low reliability when the whole group is considered, due to individual adaptations of the technique. Thus, coaches should consider that *ippon-seoi-nage*’s individual performance patterns coexist and are highly reproduced by each judoka, while at the same time, they significantly differ from the technique used by others. The rise in the jump capacity observed after the ascent to moderate altitude is not followed by changes in time, acceleration and angular velocity of the studied technique, which indicates an absence of transference. This can be due to a non-existence of transference or a sample dispersion in the use of the legs during the *ippon-seoi-nage*, indicating the need for future studies that analyse the transference between both factors. The effect of H on the reliability of the time variable linked to the leg extension in the *ippon-seoi-nage* together with the changes in the jump capacity could indicate the need to adjust and stabilize the technique after the ascent to moderate altitude.
